# Genome Evolution of a Tertiary Dinoflagellate Plastid

**DOI:** 10.1371/journal.pone.0019132

**Published:** 2011-04-26

**Authors:** Tove M. Gabrielsen, Marianne A. Minge, Mari Espelund, Ave Tooming-Klunderud, Vishwanath Patil, Alexander J. Nederbragt, Christian Otis, Monique Turmel, Kamran Shalchian-Tabrizi, Claude Lemieux, Kjetill S. Jakobsen

**Affiliations:** 1 Centre of Ecological and Evolutionary Synthesis, Department of Biology, University of Oslo, Oslo, Norway; 2 Microbial Evolution Research Group, Department of Biology, University of Oslo, Oslo, Norway; 3 Département de Biochimie, de Microbiologie et de Bio-Informatique, Université Laval, Québec City, Québec, Canada; California State University Fullerton, United States of America

## Abstract

The dinoflagellates have repeatedly replaced their ancestral peridinin-plastid by plastids derived from a variety of algal lineages ranging from green algae to diatoms. Here, we have characterized the genome of a dinoflagellate plastid of tertiary origin in order to understand the evolutionary processes that have shaped the organelle since it was acquired as a symbiont cell. To address this, the genome of the haptophyte-derived plastid in *Karlodinium veneficum* was analyzed by Sanger sequencing of library clones and 454 pyrosequencing of plastid enriched DNA fractions. The sequences were assembled into a single contig of 143 kb, encoding 70 proteins, 3 rRNAs and a nearly full set of tRNAs. Comparative genomics revealed massive rearrangements and gene losses compared to the haptophyte plastid; only a small fraction of the gene clusters usually found in haptophytes as well as other types of plastids are present in *K. veneficum*. Despite the reduced number of genes, the *K. veneficum* plastid genome has retained a large size due to expanded intergenic regions. Some of the plastid genes are highly diverged and may be pseudogenes or subject to RNA editing. Gene losses and rearrangements are also features of the genomes of the peridinin-containing plastids, apicomplexa and Chromera, suggesting that the evolutionary processes that once shaped these plastids have occurred at multiple independent occasions over the history of the Alveolata.

## Introduction

The primary endosymbiosis that gave rise to photosynthetic eukaryotes probably occurred only once and represents a major and critical event in evolutionary history. This endosymbiosis between a eukaryote and a cyanobacterium resulted in the primary plastids found in plants, green algae, red algae and glaucophytes. Subsequently, the primary plastids were laterally transferred to several eukaryotic lineages as result of a eukaryote engulfing another eukaryote carrying a primary plastid, then giving rise to secondary plastids, or even engulfed eukaryotes harboring secondary plastids (a tertiary plastid). These complex plastids are characterized by increased number of membranes (three-four) and subsequent modifications of the intracellular transport [1]–[3]. There are four major groups comprising a secondary plastid of likely red algal origin: dinoflagellates, haptophytes, cryptophytes and heterokonts.

Among these groups, dinoflagellates are unique in featuring secondary and even tertiary plastids that have independently been acquired from several algal lineages. The most common dinoflagellate plastid is distinguished by the presence of chlorophyll a and c, the pigment peridinin and three surrounding membranes. Almost half of the extant dinoflagellate species contains either this peridinin plastid, considered to be ancestral for the lineage, or have replaced it by a plastid derived from a wide range of algal lineages (including green algae, cryptophytes, haptophytes, and diatoms) [4]–[9]. The remaining dinoflagellate species have lost their photosynthetic ability and display a heterotrophic lifestyle. The variety of plastids makes the dinoflagellates the most dynamic system for acquiring and loosing plastids as well as nuclear-encoded plastid-targeted genes among eukaryotes [9]–[12] It is therefore a suitable model for studying the processes involved in plastid evolution.

During the evolution of the first primary plastids, a massive amount of the original genes present in the enslaved endosymbiont disappeared. Some of these endoymbiont genes were completely extinguished, while others were transferred to the nucleus of the new host and their products were transported back to the plastid [13], [14]. Consequently, only a few of the genes required for proper plastid function are encoded by the plastid itself. Studies of plastid genomes from chromists (haptophytes, cryptophytes, and heterokonts) have shown that these do not contain as many genes as red algal plastids [15]–[17], indicating that a process of genome and gene reduction also took place in such higher order endosymbionts. The peridinin-containing dinoflagellates have taken the reduction of their plastid genome a step further than the chromists, with only 18 genes identified so far [18]. Even more peculiar, instead of the standard circular plastid genome found in other photosynthetic eukaryotes, the peridinin-containing dinoflagellates display multiple replicating minicircles carrying one or two genes [19].

The species belonging to the *Karlodinium* and *Karenia* genera acquired their plastids via tertiary endosymbiosis. These two closely related aberrantly pigmented dinoflagellate genera have replaced their original peridinin-plastid by a plastid characterized by chlorophylls a+c and 19′-hexanoyloxy-fucoxanthin (hereafter termed fucoxanthin) [8], [20]. Phylogenies inferred from plastid gene sequences revealed that *Karlodinium veneficum* (D. Ballantine J. Larsen; previously named *K. micrum*) and *Karenia brevis* branch off as sisters within the haptophytes [8], [12], [21]–[24]. However, whether the fucoxanthin-plastid originates from a single endosymbiotic event in the common ancestor of *Karlodinium/Karenia* or from two separate events remains unknown - mainly because of the poor resolution of the reconstructed trees and because only a few haptophyte plastid genes have been analyzed so far.

Here, we have investigated how a haptophyte plastid genome is altered when the organelle is transferred to a dinoflagellate host environment. To address this, we sequenced the plastid genome of *K. veneficum* and compared the genome structure with what is known from haptophyte plastids. The results reveal major differences between *K. veneficum* and the typical haptophyte plastids, implying massive gene loss, genome rearrangements and expansion of intergenic regions. Some of these processes seem to be a reoccurring process among alveolate plastids.

## Results

### Genome assembly

The 454 sequencing resulted in 260,387 (FLX) and 625,532 (Titanium) reads, generating a total of 230 Mbp (megabasepairs) of sequence. Some *K. veneficum* plastid-encoded genes were represented by a remarkably high frequency in the 454 dataset, making the assembly of the genome complex and demanding. However, we did manage to assemble a single contig comprising 142,981 bp. The gaps between the contigs were closed by including sequences acquired from long-range PCR sequencing and 9600 genomic library clones to the assembly (see methods). Two of the PCR products that closed the gaps between contigs contained short tandem repeats (the trinucleotide motif TAA, in 21 and 113 copies, respectively). Additional PCRs for joining the contig ends failed. We are thus unable to safely conclude that the *Karlodinium* plastid genome is organized as a circle.

### Essential features of the *K. veneficum* plastid genome

**Figure 1:**
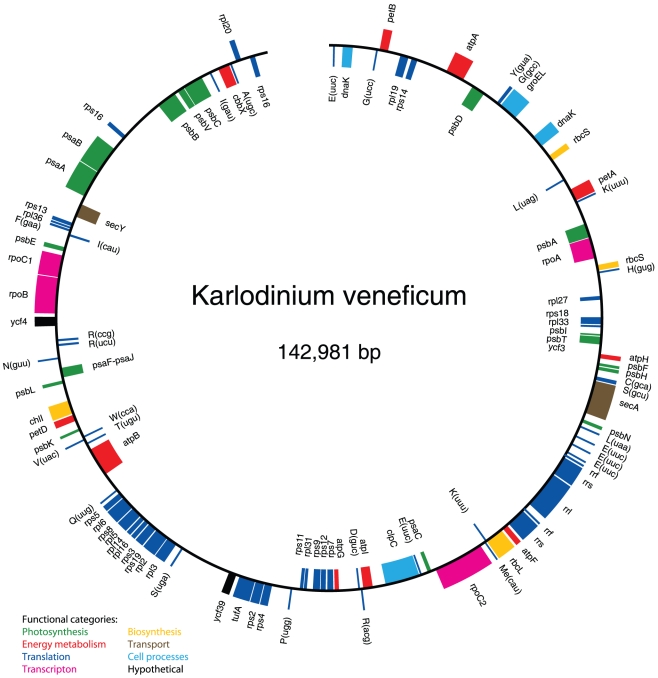
*K. veneficum* plastid genome map. Genes facing outside are transcribed in a clockwise direction, while those on the inside are transcribed counterclockwise. tRNA genes are marked with the one-letter amino acid code and the anticodon in parenthesis.

The *K. veneficum* plastid genome encodes 70 different proteins, 30 tRNAs (of which trnE and trnK is present in five and two copies, respectively) and three ribosomal RNAs. A map of the plastid genome is shown in Fig. 1 and the general features of the genome are compared with those of other chromist plastid genomes in Table 1. With an overall GC-content of 27.1%, the *K. veneficum* genome is very similar to the *E. huxleyi* genome (26.8% GC). A total of 51.3% of the *K. veneficum* genome is non-coding, and it contains 2 short putative introns of 132 and 155 bp length in trnN and trnK, respectively. Three occasions of overlapping genes were detected (rps18 and rpl33; rps8 and rpl5; psaF and psaJ, see Fig. S1), while the intergenic spacers range from 24 bp up to ∼4500 bp, with an average length of ∼730 bp. The protein-coding genes seem to use the standard genetic code and three termination codons (TAG, TAA, TGA). The most common stop codon is TAA. Two genes (groEL and rbcS) have the unusual start codon ATT. Frameshifts were found in the reading frames of five genes (rpoB, rpoC2, petD, rpl5 and secY). We could not identify an inverted repeat in the *K. veneficum* genome.

**Table 1:** General features of the *Karlodinium veneficum*, haptophyte, cryptophyte and heterokont chloroplast genomes.

	*Karlodinium veneficum*	*Emiliania huxleyi*	*Guillardia theta*	*Odontella sinensis*
Size	142,981	105,309	121,524	119,704
Inverted repeat	No	Yes	Yes	Yes
Total G+C content (%)	27.1	26.8	33.0	31.8
Gene content total	99	144	177	160
% Coding sequence	48.7	86.3	87.9	84.1
Protein coding genes (%GC)	70 (33)	113 (37)	144 (33)	128 (37)
rRNA genes (%GC)	3 (41.8)	3 (46.6)	3 (48.4)	3 (46.6)
tRNA genes (%GC)	29 (41)	27 (54.2)	27 (54.5)	27 (53.2)
No. of overlapping genes	3	1	5	4
No. of introns	2	0	0	0
Average size intergenic spacer in bp (%GC)	730	97.6	82.9	115.7
Start codons: ATG	66	105	136	123
Start codons: GTG	1	6	6	5
Start codons: other	2	None	None	None

Table modified from Oudot-Le Seqc et al. 2007.

**Figure 2:**
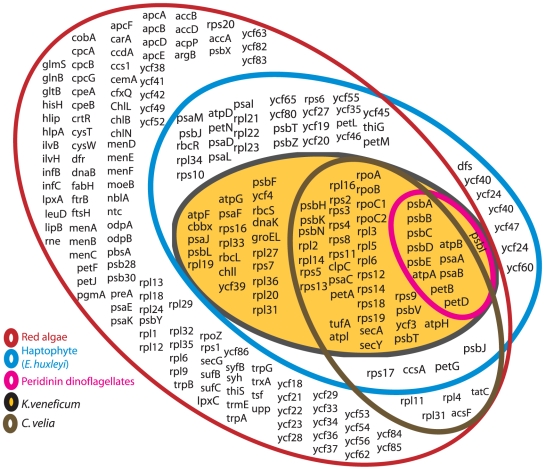
Gene content of red plastid lineages. Venn diagram of plastid genome gene content of *K. veneficum*, *E. huxleyi*, peridinin-containing dinoflagellates, *C. velia* and red algae. The *K. veneficum* plastid genome is a subset of the *E. huxleyi* genome, and there is a large overlap between the genes found in *K. veneficum* and *C. velia*. tRNAs, rRNAs and other small RNAs are not included in the diagram.

The *K. veneficum* plastid genome contains a subset of the protein-coding plastid genes found in the haptophyte *E. huxleyi* (Fig. 2), including all 12 protein-coding genes found in the extremely reduced plastid genome of peridinin dinoflagellates. The relatively low number of plastid genes in *K. veneficum* suggests that many genes were transferred from the plastid genome to the host nucleus; however, none could be identified in the available *K. veneficum* EST-library [12].

**Figure 3:**
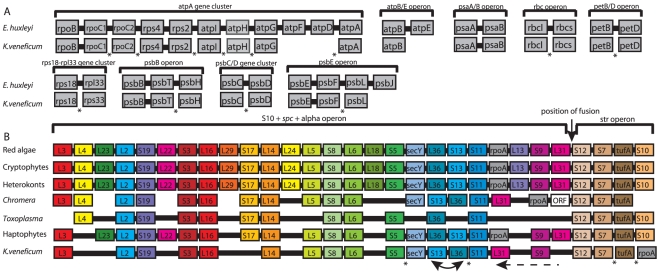
Examples of conserved gene blocks. Solid horizontal lines indicate neighboring genes. Asterisks denote that the genes are not connected. **A**: Comparison of conserved genes blocks in *K. veneficum* and *E. huxleyi*. **B**: Comparisons of the gene order in the plastid ribosomal superoperon of various red-plastid lineages. The switched order of S13 and L36 and the transition of L31 in *C. velia* and *K. veneficum* are indicated.

Gene clusters that are often conserved among chromist plastid genomes [25] are lacking in the *K. veneficum* plastid genome (Fig. 3A), including the atpA-operon and the well-known ribosomal protein superoperon, which is highly reduced and separated into five fragments (Fig. 3B). Also, the rRNA operon displays an unusual structure, where only the 5S and SSU are duplicated (Fig. 1). The figure shows the complete operon in one copy. However, our assembly is not conclusive as to whether the whole operon was present in one or two copies in addition to the truncated operon.

Several of the *K. veneficum* genes are very divergent compared to their homologues in other plastid genomes. Ten of the 70 protein-coding genes display internal stop codons (most often TAA), five require frameshifts for proper translation, five are missing large conserved segments universally found in plastid genes, and two display an extremely divergent sequence compared to their known homologs available in Genbank (Table 2, Fig. S1). These observations may be indicative of RNA editing, alternative codon usage or the presence of pseudogenes. All the internal stop codons are located within highly variable regions. It is therefore very difficult to detect a pattern indicating alternative codon usage.

**Table 2:** List of *K.veneficum* plastid-encoded genes with unusual features.

**Genes with stopcodons**	atpA, cbbx, petA, petB, psaA, psaB, rpl14, rps13, rps19, secA
**Genes that require frameshifts for proper translation**	petD, rpl5, rpoB, rpoC2, SecY
**Genes that lack large conserved segments**	atpG, ClpC, rps 11, ycf39, dnaK
**Extremely divergent genes**	atpF, rps7

### Phylogeny of the *K. veneficum* plastid genome

**Figure 4:**
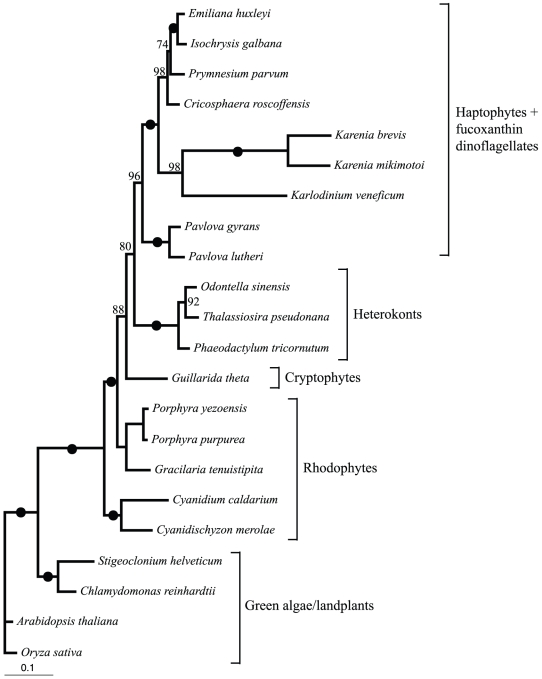
*K. veneficum* multigene plastid phylogeny. Maximum likelihood tree inferred by RAxML based on the concatenated alignment of psaA, psaB, psbA, psbC, psbD, and rbcL genes confirms the phylogenetic placement of the *K.veneficum* plastid. Dots display 100% bootstrap support.

The inferred plastid gene phylogeny shows that the *K. veneficum* and two other fucoxanthin-dinoflagellates cluster together in a monophyletic group with maximum support (Fig. 4). Together, this fucoxanthin-dinoflagellate group forms a highly supported sister group (100% BV) to the haptophyte group Prymnesiophyceae, while the other haptophyte group Pavlovophyceae branches off as a sister to the Prymnesiophyceae/fucoxanthin dinoflagellates. This topology confirms that the genome sequenced here is from a tertiary plastid of haptophyte origin [8]. In addition, it suggests that the fucoxanthin-plastids originate either from a single endosymbiotic event or alternatively several events, all involving uptake of haptophytes. Comparison of branch lengths shows that the *K. veneficum* genes evolve more rapidly than those in haptophytes and other chromalveolates (see also [8], [24]).

## Discussion

### A significant gene reduction is seen in the tertiary endosymbiont genome

The plastid genome of *K. veneficum* contains only 70 protein-coding genes, whereas the haptophyte *Emiliana huxleyi* has 110 plastid-encoded protein genes [16]. Thus, the haptophyte plastid is likely to have sustained extensive gene losses after being incorporated into a dinoflagellate. An extremely reduced gene content is characteristic for the plastid genomes of peridinin dinoflagellates; these genomes comprise the smallest number of genes of any photosynthetic lineage. Similarly, although not as extreme as in dinoflagellates, the alveolate *Chromera velia*, a photosynthetic lineage closely related to the parasitic apicomplexa also carries a gene-reduced plastid genome containing only 56 protein-coding genes [26]. This implies a major reduction of genes in several plastid-bearing alveolate lineages. Here, we show that the tendency for reduction of plastid-encoded genes also applies for *K. veneficum*, which acquired its plastid in an independent endosymbiotic event. This indicates that the property of reducing the plastid gene number is shared between the apicomplexan and dinoflagellate lineages.

Interestingly, when comparing the plastid-encoded genes of *C. velia* and *K. veneficum* a large overlap is observed: 48 of the 56 plastid protein genes in *C. velia* are found among the identified *K. veneficum* genes. This substantial proportion of shared genes indicates that the independent processes of reduction that occurred in these closely related host species led to similar gene contents, which may reflect some kind of “core” plastid genes. In our BLAST searches of expressed *K. veneficum* genes [12] against the *E. huxleyi* plastid genome, we could not identify any significant hits to the lost protein-coding plastid genes of *K. veneficum*. It is thus unknown whether these genes were transferred to the host nucleus or whether they were lost from *K. veneficum* altogether. Although the *K. veneficum* plastid genome has lost a large fraction of protein-coding genes, it has retained a nearly complete set of tRNA genes. This differs from the situation in the minicircle sequences of peridinin dinoflagellates, in which only a few tRNA genes have been identified (trnMe in *Amphidinium carterae* and trnP, trnW and trnMe in *Heterocapsa triquetra*) [27], [28]. Hence, except for trnfMet, all the genes lost from the *K. veneficum* plastid genome are protein-coding genes.

### A reshuffled plastid genome with expanded intergenic regions

In contrast to the peridinin dinoflagellates which carry their plastid genome on multiple minicircles [10], the *K. veneficum* plastid contains a large linear, or most likely, a circular chromosome. This enables us to compare the general structures of the *K. veneficum* plastid genome to that of the closely related *E.huxleyi*. Despite the reduced number of genes in the *K. veneficum* plastid genome, the sequence complexity of this genome has been substantially increased by accumulation of sequences in intergenic spacers. The intergenic regions, which are about six-fold larger (733 bp in average) than those in other chromalveolates [15], represent more than half the total genome. Thus, greatly expanded non-coding regions mainly explains the larger size of the *K. veneficum* genome relative to the *E. huxleyi* genome (143 kb vs. 105 kb) [16]. Most red algal-derived plastid genomes contain several conserved gene clusters, including the well known ribosomal protein superoperon and the atpA-operon [15]. In *K. veneficum*, however, most of these conserved gene clusters are reduced and highly scrambled compared to their closest known relatives (Fig. 3). These operons are indeed found in *E.huxleyi*, indicating that the *K. veneficum* plastid genome was reshuffled following incorporation into the dinoflagellate cell. Interestingly, the unusual relocation of rpl31 within the ribosomal superoperon is seen in both *K. veneficum* and *C. velia*, implying that this transition have happened independently in these lineages.

Together, these results show that the establishment of the tertiary plastid in dinoflagellates was accompanied by major changes to the endosymbiont genome, including expanded intergenic regions and a reshuffled gene order. Interestingly, the extensive changes seen in the *K. veneficum* plastid genome are different from the tertiary plastid genomes of ‘dinotoms’ (i.e. dinoflagellates with diatom-derived plastids). However, unlike the fucoxanthin plastid, the dinotom plastid has remained practically unchanged since the tertiary endosymbiotic event [29]. Thus, this difference could reflect the different stages of plastid integration in these two dinoflagellate lineages.

### Certain *K. veneficum* plastid genes are highly divergent

Alignments of the *K. veneficum* genes with their homologues in other plastid genomes revealed that several genes are very divergent. Some of these genes contain one or several stop codons within the assumed protein-coding sequence, indicating that the gene transcript is no longer functional, that the organism uses a deviant genetic code, or that the gene transcript is edited before translation. To determine whether the internal stop codons are due to a deviant genetic code, one may compare the same genes in close relatives and search for patterns in which a sense codon was replaced by a stop codon. We compared the *K. veneficum* genes to those of *E. huxleyi* genes but were unable identify such patterns. Thus, a deviant code is probably not the cause for the unexpected stop codons. RNA editing has been shown to be prevalent in dinoflagellate mitochondrial genomes [30]. Moreover, a few cases of RNA editing have been reported for the plastid psaA, psbB, psbE and 16S rRNA genes in *Ceratium horridum* [31], for psbA in *Lingulodinium polyedrum* [32] and for eight of 10 examined plastid genes in *Heterocapsa triquetra* [33]. Based on the documented cases of RNA editing in dinoflagellate organelles, we favor the idea that most of the divergent *K. veneficum* genes are subject to RNA editing. However, the possibility that the genetic code is deviant, or that some of the genes with unusual features are pseudogenes cannot be excluded.

### Serial endosymbiosis events as a driving force in dinoflagellate plastid evolution?

The *K. veneficum* plastid genome is a result of tertiary plastid replacing a previous secondary plastid. Dinoflagellate lineages that have undergone plastid replacements, including *Lepidodinium chlorophorum* (green plastid) and *Karenina brevis* (haptophyte-derived plastid) have been shown to contain a phylogenetic mosaic of plastid targeted, nuclear encoded genes reflecting the previous photosynthetic evolutionary history of the species [9], [11]. The evolutionary adaptation from one functional plastid to another – including reuse of some of the genes from the previous endosymbiont – is likely to accelerate, rather than conserve, evolution of the plastid-encoded genes of the new endosymbiont. To what extent the divergent plastid genome of *K. veneficum* is a result of processes associated with adaptation of the new endosymbiont to an evolutionary mosaics of the plastid-targeted genome, remains to be investigated.

## Materials and Methods

### Culturing

A *K. veneficum* culture (UIO083) obtained from the algal collection at the Department of Biology, University of Oslo, Norway, was grown in ½ × IMR medium [34] prepared from filtered seawater. Bacteria were removed from the culture by antibiotic treatment using a mixture of penicillin, streptomycin and gentamycin (100/50/50 mg/L respectively) and one drop of bacteria-test medium (½ × IMR medium supplemented with 1 g trypton and 0.25 g yeast extract per L) for 94 h. The culture was tested for the presence of bacteria using bacteria-test medium and flow-cytometry. Both methods tested negative for bacteria, confirming that the culture was most likely axenic. The culture was grown with air bubbles in 10 L bottles and harvested using a flow-through centrifuge. The cells were snap-frozen in liquid nitrogen, freeze-dried and stored at −80°C.

### Plastid DNA purification and sequencing

Total cellular DNA was extracted and centrifuged in CsCl-bisbenzimide density gradients according to the method described in [35]. Each gradient was fractionated into 40 fractions using a Density Gradient Fractionation System (Brandel, Gaithershurg, MD). The fractions containing plastid DNA were identified by Southern blot analyses as follows. Aliquots of all fractions were cut with EcoRI and size-fractionated on an agarose gel, and then a blot of the gel was hybridized with a ^32^P-labelled fragment specific to the *K.veneficum* rbcL, psbA, psaA or 23S rDNA. Each probe was prepared by random labeling of a purified plasmid DNA insert with the NEBlot kit (New England Biolabs). In each gradient, positive hybidrization signals were located to five or six AT-rich fractions containing a very low amount of DNA (<250 ng). These fractions were pooled and used as a template in rolling circle amplification (RCA) reactions using the Repli-g kit (Qiagen Inc., Mississauga, Canada) [36]. The RCA-amplified, plastid DNA was sheared by nebulization to produce fragments of 1.5 to 2.5 kb that were subsequently cloned into pSMART-HCKan (Lucigen Corporation, Middleton, WI). A total of 9600 plasmid clones were picked manually and hybridized with the original plastid DNA used as a template in the RCA reaction. DNA templates from positive clones were prepared with the QIAprep 96 Miniprep kit (Qiagen Inc., Mississauga, Canada) and sequenced at Laval University or at the University of Oslo by Sanger sequencing, using universal primers. Sequences were edited and assembled using Sequencher 4.8 (GeneCodes, Ann Arbor, MI).

### 454 Pyrosequencing

The Sanger sequencing of individual plasmids did not yield sufficient coverage to assemble the *K. veneficum* genome. We therefore performed two runs of 454 pyrosequencing [37]. Two different samples of plastid-enriched DNA were used as a template for the 454 sequencing (one that had been RCA-amplified as described above, and another that had not been subjected to RCA). Each sample consisted of pooled fractions. Pyrosequencing was performed on a GS FLX (Roche, 454) at the Norwegian Sequencing Centre (http://www.sequencing.uio.no), according to the manufacturer protocols. The resulting reads were assembled using Newbler v2.3 (gsAssembler), using default settings and Celera v.6.1 [38] with following parametres: overlapper = mer, unitigger = bog, utgErrorRate = 0.03. The resulting contigs were imported into Sequencher 4.8 and assembled together with the sequences obtained from the clone library. Long range PCR was performed to fill gaps between the assembled contigs, using the enzyme BD Advantage (Clontech, CA, USA) on a DNA template isolated directly from the *K. veneficum* culture (primer sequences provided in Table S1). The final contig was parsed, using a custom PERL script, to obtain the per-contig read depth and consensus sequences as per [39]. Genes, ORFs and RNAs were identified either as described by [40] or by ORF Finder, BlastX and BlastN searches against the database at the National Center for Biotechnology Information (http://blast.ncbi.nlm.nih.gov/). Transfer RNA genes were identified using tRNAscan-SE v.1.21 [41].

### Phylogenetic analyses

Alignments of the amino acid sequences encoded by psaA, psaB, psbA, psbC, psbD, and rbcL were constructed for phylogenetic analyses. The alignments included sequences from *K. veneficum* and representatives from all chromist lineages. Maximum likelihood analyses of the single protein alignments, as well as the concatenated dataset were performed using the program RAxML on the Bioportal at University of Oslo (www.bioportal.uio.no), using the PROTMIXWAG amino acid substitution model. 100 separate phylogenetic analyses from random starting trees were run. Bootstrap support was inferred using 100 pseudoreplicates with the same model as in the original analyses.

## Supporting Information

Figure S1 **Genes with unusual features.** Genes with unusual features in the *K. veneficum* plastid genome.
(EPS)

Table S1 **List of primers.** Primers used in PCRs for filling gaps between contigs.
(DOC)
