# Supplementary material for: Genome Evolution of a Tertiary Dinoflagellate Plastid
Source: PLoS One. 2011 Apr 26;6(4):e19132. doi: 10.1371/journal.pone.0019132 (PMC3082547; doi:10.1371/journal.pone.0019132)
Supplement: Table S1 — List of primers. Primers used in PCRs for filling gaps between contigs. (DOC) [file pone.0019132.s002.doc]

Supplementary Table S1. PCR primers used for outwards directed amplification to close gaps between master genome contigs.

| Primer name | Primer sequence | PCR product size | Accession number |
| --- | --- | --- | --- |
| C0010_687out  C0014_592out | AGCCATGCATATGCAAGAACAG  AATGAGAGGTTCTTAGCGCGTG | 1667 bp |  |
| C0014_21576out  C0160_11140out | TATCGTATCTCACATGCGTAGG  GTCTAGAGGTAGCGCATTGCTC | 889 bp*) |  |
| C0160_254out  C0001_873out | AGGCTAATATCGTAGGTGAGAC  TCCGAGTAGATTTTGCACTAGC | 1093 bp |  |
| C0015_575out  C1712_919out | ACTCGCTTAGGAGATAGACAGC  AATAGAGTGACAAGTATACGAG | 1469 bp**) |  |
| C0008_12705out  C1694_911out | TAAGCTTAGAAAGTCACCGTAG  CGTCATTGCTTTTGTGTGGCAG | 5762 bp |  |

*) Repeating motif, ca. 113 copies of the motif TAA

**) Ca. length, long mononucleotid stretches
